# Designing Implementation Strategies for a Digital Suicide Safety Planning Intervention in a Psychiatric Emergency Department: Protocol for a Multimethod Research Project

**DOI:** 10.2196/50643

**Published:** 2023-11-09

**Authors:** Hwayeon Danielle Shin, Juveria Zaheer, John Torous, Gillian Strudwick

**Affiliations:** 1 Institute of Health Policy, Management, and Evaluation, University of Toronto Toronto, ON Canada; 2 Krembil Centre for Neuroinformatics, Centre for Addiction and Mental Health Toronto, ON Canada; 3 Health Outcomes and Performance Evaluation (HOPE) Research Unit, Institute for Mental Health Policy Research, Centre for Addiction and Mental Health, Ontario, Canada Toronto, ON Canada; 4 Gerald Sheff and Shanitha Kachan Emergency Department, Centre for Addiction and Mental Health Toronto, ON Canada; 5 Department of Psychiatry, University of Toronto Toronto, ON Canada; 6 Department of Psychiatry, Beth Israel Deaconess Medical Center, Harvard Medical School Boston, MA United States

**Keywords:** implementation science, suicide prevention, eHealth, mental health, health informatics, integrated knowledge translation, co-design, research protocol, mobile phone

## Abstract

**Background:**

Suicide prevention is currently a national health priority in Canada. Emergency departments (EDs) are critical settings for suicide prevention, and in our local psychiatric ED at the Centre for Addiction and Mental Health, we plan to embed an app-based tool called the Hope app to support suicide safety planning intervention. The app is free and available on app stores, and usability tests have been completed. As a next step to embed this new tool into the routine clinical workflow, research is needed to assess determinants of and design strategies for implementation with the end goal of routinization.

**Objective:**

The purpose of this 2-phased research is to implement the app in the routine clinical workflow in our local psychiatric ED. The specific objectives are as follows: (1) understanding ED clinicians’ perceptions and experience of implementing the app in routine practice and identifying barriers to and facilitators of implementation (phase 1) and (2) using findings and outputs from phase 1 and collaborating with service users, families, and ED clinicians to co-design implementation strategies for the app (phase 2).

**Methods:**

We will use an integrated knowledge translation approach throughout this project. In phase 1, we will conduct interviews with ED clinicians to identify implementation determinants using a behavior change framework. In phase 2, a co-design team comprising clinicians, ED service users, and families will design implementation strategies that align with the determinants identified in phase 1.

**Results:**

This protocol presents detailed information about the entire structure of the 2-phased research project. Ethics approval for conducting the qualitative descriptive study (phase 1) has been obtained, and the recruitment and data collection processes will be completed no later than December 2023. Ethics approval for phase 2 is underway.

**Conclusions:**

Involving multiple knowledge user groups early in the research and decision-making process is crucial for successful implementation. Although co-designing is commonly practiced during innovation development, there is often a misconception that the responsibility for implementing what has been designed falls on others. This research aims to fill this methodological gap in the health informatics literature. By the end of this project, we will have developed theory-informed implementation strategies to support Centre for Addiction and Mental Health ED clinicians in adopting the Hope app to complete safety planning intervention. These strategies, guided by a behavior change framework, will target clinicians’ behavior change and seamlessly integrate the app into the routine clinical workflow. In addition, this research project will provide recommendations on how to involve multiple knowledge user groups and offer insights into how the methodology used can be adapted to other areas within the health informatics literature.

**International Registered Report Identifier (IRRID):**

PRR1-10.2196/50643

## Introduction

### Background

Suicide is currently the second leading cause of death among youth in Canada [1]. Emergency departments (EDs) are critical settings for suicide prevention for many reasons [2]. First, individuals, both adult and pediatric patients, requiring mental health services visit EDs more frequently than those who do not [3-5]. Second, EDs frequently provide care to individuals who attempt suicide and are often the first point of contact for individuals who experience suicidal crises [2,6,7]. Third, when looking at service use patterns for people who died by suicide, many of them were already in contact with health services, including EDs [8-10]. For example, a Canadian study published in 2014 examined 8851 suicide deaths in Quebec and found that one-third of these individuals had visited an ED within the month before death [9]. Finally, some individuals living with suicide-related thoughts and behaviors seek help in the ED as a last resort [11,12]. Although this indicates a promising fact that individuals are continuing to seek help, suicide risk may not always be obvious, recognized, or addressed [13-18]. As such, there is a critical need for research to bridge the evidence-to-practice gap in suicide prevention practices within ED settings, and EDs present a critical window of opportunity for identifying and providing care to patients at risk of suicide.

Currently, safety planning intervention (SPI) [19] is one of the best available practices for suicide prevention [20,21]. There is an evidence base (ie, systematic reviews) to support the effectiveness of SPI in reducing suicidal behaviors [20,21]. For example, SPI has been shown to decrease suicidal behaviors by up to 45% [22]. SPI is a collaborative process between clinicians and patients for developing a plan regarding coping strategies, emergency contacts, and restriction of lethal means [19]. SPI is traditionally delivered pen-on-paper, which limits portability. Patients report trouble locating or losing the paper safety plan [23,24], which can be a dire challenge during crises as SPI cannot generate its intended benefit given that it is not available *at hand*. Studies have shown that mobile phones as an intervention modality are acceptable, feasible, and useful for mental health patients [25-27], including those managing suicide-related thoughts and behaviors [28,29]. Moreover, the use of apps and various forms of information and communications technologies (ICTs) has the potential to enhance suicide prevention efforts through the elimination of geographical limitations while also increasing the accessibility and availability of evidence-based interventions [30].

At the Centre for Addiction and Mental Health (CAMH) in Canada, a research team collaborated with clinicians and patient partners and developed an app-based tool for SPI called the Hope app by CAMH [31]. In 2019, at the Innovation Expo held at CAMH, the Hope app was introduced as an innovative solution [32,33]. The idea for the app was presented by an individual with lived experience who had cared for a family member who had attempted suicide [32,33]. The Hope app has several strengths, with its primary advantage being its accessibility “at hand” for individuals living with suicide-related thoughts and behaviors. The idea of having a suicide prevention tool readily available was the main reason behind its recognition at the Innovation Expo [32,33]. In addition, the app provides other valuable resources, including the ability to email the safety plan. It is well documented in the literature that friends and families play a significant role in helping individuals who are seeking assistance when dealing with suicidal thoughts and behaviors [34-39]. As suicide-related symptoms worsen and suicidal thoughts and behaviors become more frequent, friends, families, or other trusted individuals can offer emotional support and encourage professional help if necessary [34-39]. The Hope app’s feature of sending a safety plan is a valuable tool for patients to inform their close social network.

The Hope app is free on app stores and is compatible with Android and Apple phones. A usability test of the app was completed at the CAMH, and a total of 54 REDCap (Research Electronic Data Capture; Vanderbilt University) surveys were administered [40]. Among the respondents, 57% found wellness activity content in the app, such as grounding techniques, to be the most frequently used, and 67% reported that the app increased their knowledge of safety planning [40]. Second, a total of 10 usability participants were recruited, and the research team administered the System Usability Scale, a validated 10-item questionnaire [41]. This yielded an overall usability score of 88.2 out of 100, with higher scores indicating better usability [40]. To better understand users’ perspectives, 18 interviews were conducted [40]. It was found that the app was helpful in prompting self-reflection, which made the safety planning process easy [40]. For instance, the paper-based safety plan used at the CAMH requires patients to provide their own answers for coping strategies, whereas in the app, users are presented with potential strategies and can choose relevant ones or add text if the provided options do not align with their needs. In addition, interview participants reported that the app is a useful tool to accompany mental health appointments [40]. Finally, user feedback was obtained from a public avenue at the Apple App Store “Ratings and Reviews” section [42]. As a next step, theory-informed research is needed to develop implementation strategies to ensure the successful delivery of the Hope app to patients who are at risk of suicide.

Implementation in health services research is about promoting the systematic uptake of evidence-based practices into routine care [43-45]. The systematic uptake of evidence-based practices is dependent on the behaviors of individuals who are involved in health services, including clinicians [46,47]. Although changing behavior is challenging, it is most effectively achieved when behavior change strategies are informed by evidence-based principles and theories of behavior change [48-50]. New innovations or ICTs such as the Hope app do not implement themselves. Implementation requires “organizational effort directed toward diffusing [ICTs] within a user community,” with the end goal of routinization [51]. Implementation efforts may include training, making changes in the clinical settings and workflow, providing technical support, or clarifying professional responsibility related to an innovation, all of which are determinants of behavior change influencing one’s knowledge, motivation, and opportunity [52]. The organizational decision to adopt the Hope app was based on existing evidence of SPI effectiveness and the organization’s priority goal for suicide prevention. As mentioned previously, there are existing systematic reviews supporting the effectiveness of SPI in reducing suicide behaviors [20,21]. Although ICT-based interventions for suicide prevention are still in their early stages, they show promise. For instance, a meta-analysis of these interventions demonstrated a small but significant efficacy in reducing suicide ideation [53]. However, one could argue that, owing to the limited clinical integration of apps [54], the current literature lacks evidence to support the effectiveness of SPI *when* it is delivered via an app (ie, changing the mode of delivery) [55]. This question of concern falls beyond the scope of our study and is an area for future investigation. Nonetheless, the findings from our study can inform the feasibility of conducting a larger experimental design to assess the effectiveness of the Hope app and facilitate its initial integration into routine clinical practice.

EDs are a critical setting for suicide prevention [56], which makes them a priority setting for the implementation of the Hope app. Implementation occurs after organizations make decisions to adopt a new innovation [51], and the end goal of implementation—routinization—involves changing clinicians’ behavior [49]. Given the organizational-level decision at the CAMH to adopt the Hope app, this proposed research will concentrate on the subsequent stages of implementation; these stages include adaptation, acceptance, and routinization [51], primarily focusing on changing clinicians’ behavior. Adaptation is about developing and revising departmental procedures, such as making changes to the workflow or training staff for new ICTs [51]. Acceptance is about promoting staff’s commitments to using new ICTs, and finally, routinization means integrating new ICTs into a normal workflow [51].

### Problem and Rationale for This Study

To date, research on mental health apps lacks rigorous implementation and a theoretical basis [54]. In addition, a recent call was made for research to consider the complex contexts in which apps are being implemented to tailor implementation efforts [57]. Without a careful investigation of the context, implementation strategies have often been mismatched with existing barriers in a given context [58,59]. As a prior work for this study, Shin et al [52] conducted a scoping review of ICTs (eg, apps) for suicide prevention that have been implemented in clinical settings and identified that barriers and facilitators collectively influence clinicians’ capability, opportunity, and motivation to implement innovations. Therefore, implementation strategies must be tailored and multifaceted to simultaneously target these determinants of behavior change to maximize implementation [60]. However, most of the reported implementation strategies focus on improving knowledge and building skills (ie, capability) without addressing clinicians’ motivation and opportunity [52]. The fact that implementation strategies are often developed nonsystematically and do not match contextual implementation determinants is a consistently reported gap in the literature [61].

Another concern with implementing a new innovation of this type is that not everyone owns or has access to a smartphone and unlimited internet or data or has digital literacy skills [62]. Inequities in accessing and using ICTs, as well as the associated outcomes of using ICTs, constitute a phenomenon called the digital divide [63]. There are three levels to the digital divide: (1) access to ICTs and necessary connections, (2) literacy to use these ICTs, and (3) capacity to convert the use of ICTs into outcomes [64]. The digital divide is rooted in social factors such as education, income, age, and place of residence [63,65,66]. To prevent digital divide amplification and avoid unintended harm, implementation strategies must be developed with digital equity considerations in mind [67]. This research is not sufficient to address the structural factors that perpetuate the digital divide but will consider its second and third levels. For example, we will adopt a set of recommendations made by Shaw et al [67] to promote digital health equity in the context of implementing ICTs in health care, such as supporting patients with low digital literacy, collaborating with service users, and providing alternatives (eg, traditional paper format of SPI and emailing electronic copies of safety plans). In addition, the interview guide and co-design session goals in this study include prompts related to digital equity.

### Research Objectives

To implement the Hope app with digital equity considerations, this proposed research aims to leverage the integrated knowledge translation (IKT) approaches as well as implementation science frameworks to develop tailored implementation strategies in a psychiatric ED at the CAMH. This project has two main objectives with two corresponding phases, as follows:

1. Understand clinicians’ perceptions and experiences of implementing the Hope app in routine practice in the psychiatric ED and identify barriers to and facilitators of implementation (phase 1)
2. Use the findings and outputs from phase 1 and engage with knowledge users to co-design implementation strategies for the Hope app (phase 2).

## Methods

### Theoretical Frameworks

There is evidence strongly suggesting that theory-informed strategies are more likely to be effective in changing behavior [48-50]. A combined use of theories, models, and frameworks is appropriate to meet the needs of researchers [68]. In this study, several frameworks will be used to address the research objectives.

One way to solve complex problems is to work with knowledge users [69] who will be delivering or receiving the Hope app. Knowledge users are people who will be using research findings to inform their decision on practice, policies, and programs, as well as people who will be affected by research findings and making informed decisions based on them [70]. As such, knowledge users include but are not limited to health care professionals, service users, and health care managers [70]. Collaborative research approaches such as IKT are perceived to be the best ways to produce relevant research outputs to address health care issues and generate a greater impact on practice [71,72]. Although co-designing is common for innovation development, people often think that implementing what has been designed is the responsibility of others [73]. This is not true; researchers can co-design changes in the workflow to support implementation [74].

IKT approaches will be used throughout this proposed research. Specifically, a medical head of the CAMH ED and a CAMH nursing chief clinical informatics officer are coauthors of this paper and members of the steering committee who will oversee the current project throughout phases 1 and 2, ensuring that the research objectives and outputs are relevant to the implementation setting. Securing leadership support for this project is an important facilitator for maintaining commitment to coproduction, as indicated in the IKT literature [71,75]. Patient partners (ie, ED service users) are also a critical knowledge user group, but they will not be part of the steering committee. However, we consulted the CAMH Lived Experience Advisory Group in February 2023. Feedback was received and has been incorporated into this protocol. Such feedback includes co-design team formulation, consensus-achieving activities, and strategies to mitigate dropout. In addition, patient partners and families or caregivers will be closely involved as co-designers in phase 2.

The Knowledge-to-Action (KTA) cycle [76] is a process model that outlines the steps and activities required to integrate a new innovation into practice [48]. We are using KTA to guide the overall research. Specifically, the research objectives have been guided by the KTA action phases leading to effective implementation. Not all stages of KTA will be addressed in this research, and this flexibility of KTA application is one strength that can fit researchers’ needs. The objectives of this research will address the following 3 steps of KTA: adapt the identified knowledge or research to the local context; assess barriers to using the knowledge; and select, tailor, and implement interventions to promote the use of knowledge. A previous study addressed steps 1 and 2 of KTA, in which we used a scoping review methodology to explore the breadth of existing evidence on ICT-based interventions for suicide prevention and collated identified implementation characteristics [52]. This step was necessary to make sense of all the relevant knowledge and identify gaps that require new research. Phase 1 in this project will then address steps 3 and 4 using a qualitative design to investigate barriers and facilitators specific to our implementation setting. Phase 2 will then address step 5 selecting and tailoring implementation strategies using a co-design approach to address barriers and leverage the identified facilitators.

KTA outlines steps to translate knowledge into practice, but it does not inform how we can achieve these steps. Therefore, we will use the Behavior Change Wheel (BCW) [77] and the Theoretical Domains Framework (TDF) [78] to further guide this research. The BCW and TDF are comprehensive and evidence-based behavior change frameworks. The BCW was developed from a synthesis of 19 behavior change frameworks. The BCW comprises multiple components, and at its core, it presents the Capability, Opportunity, and Motivation–Behavior (COM-B) model, which is an implementation theory that explains the relationships between internal and external influences of behavior change [46,77]. The TDF is often used as a supplement as it provides a granular breakdown of the COM-B [79]. The BCW can assist with barrier and facilitator assessment and inform the design of implementation strategies [46,77]. The BCW also offers step-by-step guidance on making theory-informed choices of strategies that link to the determinants of behavior change based on the barrier and facilitator analysis [46,77].

There are several related tools that augment the use of the BCW. The Affordability, Practicability, Effectiveness, Acceptability, Safety, and Equity (APEASE) selection criteria are a tool to support decision-making [46,77]. As the BCW provides a wide range of options, it may not be feasible to leverage all possible intervention options. This is when the APEASE criteria can be used to rank options and carefully select the top choices based on the knowledge users’ assessment of options considering all domains of APEASE. Next, the behavior change technique (BCT) Taxonomy version 1 provides a granular level of the 9 intervention types [80]. BCTs are the smallest unit and the “active ingredients” of an intervention that generate behavior change [80]. The development of the taxonomy was an international consensus project, and it provides a common language between researchers and allows for clear communication and replication of interventions [80]. Finally, the Theory and Technique Tool provides information on linkages between mechanisms of action (ie, how behavior can be changed) and specific BCTs [81]. It is based on the synthesis of evidence for the links, expert consensus, and triangulation of these 2 sources of findings [82]. This tool provides links between 74 BCTs and 24 mechanisms of action (ie, COM-B) to change behavior [81].

Lynch et al [83] suggest using the BCW and TDF when researchers investigate individual experiences as a preparation for implementation. This is because clinicians’ behavior is a determinant of implementing new ICTs, and therefore, it is vital to conduct a thorough behavioral analysis, such as barriers to and facilitators of performing the desired activity. In this study, our desired behavior change for the clinical team is to deliver the SPI using the Hope app on patients’ own devices. The BCW and TDF have been used in a wide range of disciplines such as health promotion [84-87] and health informatics [88-90] to characterize barriers to and facilitators of implementation. For example, in the field of health informatics, the BCW and TDF have been used to inform implementation strategies to facilitate the completion of electronic discharge summaries [90].

### Study Setting

This study will take place in the CAMH Gerald Sheff and Shanitha Kachan ED located in an urban academic tertiary care center in Ontario that provides care to approximately 1200 adult patients each month [91]. The CAMH ED is Ontario’s only stand-alone psychiatric ED that provides emergency assessment and treatment exclusively focused on adults (aged ≥16 y) with mental health and substance use issues. Approximately 150 staff members (both full time and part time) are employed in this ED. On a daily basis, the ED is staffed by a total of 16 to 20 nurses, program assistants, psychiatrists, residents, and social workers.

### Intervention: Hope App

The Hope app by CAMH is a free smartphone app that provides suicide prevention information, resources, and tools to support individuals who are experiencing suicide-related thoughts and behaviors. This app is evidence-based, meaning that it adheres to the best practices available for safety planning [20,21], and was developed in collaboration between service users and clinicians. The collaborative approach to developing the Hope app is a strength, and it has been well documented in the literature that research outputs (eg, interventions) generated through partnerships are more useful and relevant to end users [92]. A core component of the Hope app is a “Safety Plan” (ie, SPI) that includes five items for completion: (1) warning signs, (2) reasons for living, (3) coping strategies, (4) support network, and (5) environmental safety. Multimedia Appendix 1 provides screenshots of the app and its content.

In addition to the SPI, the app includes a direct 9-1-1 line, mental health resources (eg, link to community resources), and wellness activities (eg, journaling and relaxed breathing). This app does not track the user’s location and cannot store or access personal data such as names, addresses, and phone numbers. Only when users want to can they share their safety plan with their clinicians or social network via email. The Hope app allows users to send an email copy of the safety plan using the default email app, and users can edit the subject or body of the email before sending it.

### Phase 1

In phase 1, we will conduct a qualitative descriptive study [93] to understand clinicians’ experiences and perspectives of implementing the Hope app in routine practice in the psychiatric ED and identify barriers to and facilitators of implementation (objective 1).

#### Sampling and Recruitment

We will use purposeful sampling strategies to recruit a heterogeneous sample to obtain broad insights and information-rich data [94]. All CAMH ED clinicians (ie, physicians, nurses, social workers, and program assistants), including trainees such as residents, will be invited for an interview regardless of their age, gender, and years of experience. The composition of the sample will be purposeful to maintain sufficient representation from each clinician group (ie, at least 2 members/discipline). Nurses make up the majority clinician group in the CAMH ED, so the sample may have a large proportion of nurses. Since the launch of the Hope app without systematic effort for integration, some staff members have been voluntarily using the app, whereas others continue to use the paper-based safety plan or a mixture of both. All clinicians will be invited whether they use the app or not. Both perspectives (ie, users and nonusers) will be valuable to gain insights into adopting the Hope app in routine practice. A specific number of participants will not be predetermined, but instead, data collection and analysis will occur concurrently such that recruitment will stop after data saturation (ie, information redundancy) [95]. Qualitative descriptive studies tend to have an average sample size ranging from 20 to 35 [96]. However, based on a systematic review of empirical tests assessing the number of interviews needed for data saturation, 9 to 17 interviews are sufficient for achieving data saturation [97]. As such, it is possible to reach saturation with <20 interviews. In short, phase 1 aims to recruit a minimum of 20 interview participants, but it could potentially be <20 if data saturation is achieved. Snowball sampling may be used as a secondary method if faced with recruitment challenges using posters and emails.

#### Data Collection

A semistructured 1:1 interview guide was informed by the COM-B and TDF (Multimedia Appendix 2). The guidelines provided by Atkins et al [79] and Michie et al [77] on applying the COM-B and TDF in research processes and example interview questions to assess each domain of the COM-B and TDF were reviewed. In addition, published examples by other researchers on how to phrase questions to assess all the domains in the COM-B or TDF were reviewed [98-100]. Through this review, adaptations were made to our interview guide to better fit our research context. In addition, a few prompts were added related to digital equity concerns. For example, clinicians will be asked whether they have encountered any challenges while using the app with individuals who have low digital literacy and faced difficulties in providing technical support to these individuals and their thoughts on the potential drawbacks of using either the paper or app format of SPI. In particular, the provision of technical support to patients in the ED aligns with the recommendation by Shaw et al [67] to integrate supportive intermediaries. However, this needs to be explored in the CAMH ED for its feasibility and for redefining professional responsibilities as the role of providing technical support may or may not be perceived as a clinical responsibility of the ED team. Open-ended questions in the interview guide are mapped according to the COM-B and TDF domains, and they are designed to identify what clinicians need, what they are capable of, and what their motivation is for using the Hope app in their normal clinical workflow. In addition, the sustainability potential of the Hope app within the CAMH ED and scalability beyond the CAMH ED will be explored. The interview guide will be pilot-tested with the internal research team to minimize any ambiguity in wordings. All interviews will be 45 to 60 minutes in length, via web-based video call, telephone, or in person, at a suitable time for the clinicians. Recognizing that accessing interviews with clinicians is challenging, we will need to be flexible with the interview format, setting, and timing and strive for conversation-like interviews, which are less formal in structure. For instance, interviews may be initiated on-site at the CAMH ED, and the remainder of the questions may be completed off-site. Questions that can be asked on-site (eg, questions 2, 3, 9, and 17 in Multimedia Appendix 2) will aim to highlight “in the moment experience” [101] in the ED context as it relates to SPI with the Hope app. All interviews will be audio recorded and transcribed verbatim and have identifying information removed to maintain privacy. We will also collect participant characteristics (eg, age, sex, gender, discipline, and years in practice) at the end of each interview via a web survey using REDCap [102]. Demographic and interview data will not be linked.

#### Data Analysis

Participant demographic characteristics will be tabulated (ie, descriptive statistics) and narratively described. Interview transcripts will be analyzed using directed content analysis [103] for barriers and facilitators using the COM-B and TDF as a codebook along with operational definitions for all domains. First, 2 reviewers will read the transcripts to categorize relevant statements into the COM-B domains and subcategories of the 14 TDF domains. These categorized segments, known as coding stripes, will be compared between the 2 reviewers for consistency. Once all transcripts are initially coded into COM-B and TDF domains, we will generate specific belief statements in relation to the 14 TDF domains. The belief statements, or initial themes, are a group of similar responses that suggest a potential influence on the target behavior [79]. These belief statements will then be further refined into themes and aligned with the COM-B domains. The final themes organized into COM-B domains will represent the barriers and facilitators perceived to influence clinicians’ behavior when delivering the SPI using the app downloaded on the patients’ own devices in the ED.

As advised by Atkins et al [79], it is important to emphasize that, although the TDF domains are intentionally designed as broad groupings of potential factors influencing behavior, the primary goal is to examine these domains in greater depth. Some statements from the transcripts may overlap across multiple domains, illustrating the intricate nature of human behavior [79]. The main domains of behavioral influences have been identified a priori, but there may be new behavioral factors unique to the CAMH ED and the Hope app. Therefore, new codes and categories may emerge during the categorization step [103]. At least 2 researchers will first trial the codebook with 1 or 2 transcripts to ensure consistency in the coding strategy. We then will independently code all transcripts. The research team will meet regularly to resolve any interpretation discrepancies through discussion or with a third person. During these regular meetings, we will also look for repetition in participants’ responses to determine whether data saturation has been reached. We will use the NVivo software (Lumivero) [104] for data management and analysis. A summary of the findings will be presented via web meetings or email communications to participants for member checking [105] to determine whether they feel that the identified themes are accurate. Outputs from phase 1 will include a list of implementation barriers and facilitators mapped to the COM-B.

#### Strengths and Limitations

Barriers and facilitators do not linearly affect clinicians’ behavior and subsequent implementation. The clinical context as well as human behavior are complex; it is not individual factors that facilitate the implementation of a new innovation but the dynamic interaction between them [106,107]. Nonetheless, the COM-B accounts for interactions between both internal (ie, capability and motivation) and external (ie, opportunity) factors that influence behavior change. Data analysis using the COM-B and TDF as guiding theoretical frameworks will help explain the relationship between factors that influence clinicians’ behavior leading to delivering the Hope-app. Therefore, the application of the COM-B and TDF will not downgrade the complexity of human behavior but, rather, will help us organize the data in a way that makes them accessible to work with and facilitates the understanding of complexity.

Phase 1 is limited to clinicians’ perspectives, but other ongoing studies at CAMH focusing on patients’ perspectives on SPI can be leveraged. Furthermore, we will involve patients (ie, ED service users) in the subsequent phase 2 to co-design implementation strategies with clinicians to integrate the Hope app in the CAMH ED. Patients’ insights will be sought during phase 2, and there will be opportunities to identify convergence with and divergence from clinicians’ perspectives.

### Phase 2

#### Overview

**Figure 1:**
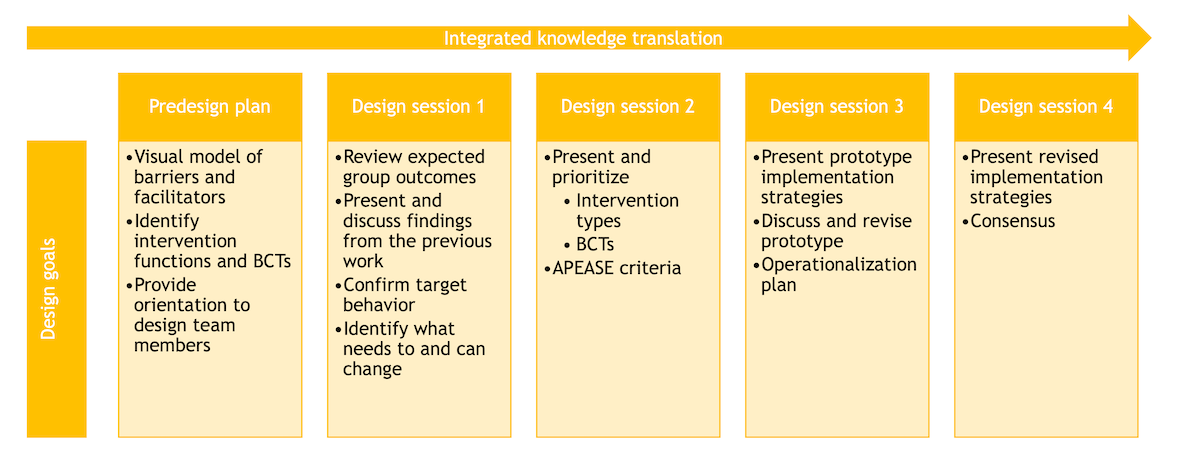
Proposed co-design process informed by the Behavior Change Wheel. APEASE: Affordability, Practicability, Effectiveness, Acceptability, Safety, and Equity; BCT: behavior change technique.

In phase 2, we will use a co-design approach to develop implementation strategies based on the outputs from phase 1. Specifically, we will select BCTs (ie, active ingredients of implementation strategies) that can mitigate barriers and enhance facilitators. The development of implementation strategies is envisioned in at least 4 co-design sessions, with specific design goals guided by semistructured, open-ended questions, and the findings from each session will feed into the next. All 4 co-design sessions will follow the steps (Figure 1) based on the BCW guide for intervention design and recommendations for co-design [108,109].

#### Sampling and Recruitment

A co-design team will consist of ED service users (ie, patients), families, and ED clinicians. The representation of clinicians on a team will include at least one nurse and one psychiatrist currently working in the CAMH ED. The composition of the co-design team will be purposely chosen to maintain sufficient representation from each knowledge user group and ensure adequate peer support for patients and families in each co-design session. At least 6 members (ie, 2 service users, 2 family members, and 2 clinicians) but no more than 10 will form a co-design team. The team will ideally stay the same throughout phase 2, facilitating rapport building. However, time and availability of participants may lead to challenges in recruitment and retention, so snowball sampling may be used as a secondary method.

#### Design Procedures

##### Overview

All co-design sessions will last approximately 1.5 to 2 hours, either on the web or in person depending on participants’ preferences and convenience. To minimize attrition, we will provide a tailored orientation to prepare all participants before each design session for effective collaboration. During this preparation, we will ensure that participants’ level of knowledge of the Hope app and the purpose of phase 2 are the same, ensuring that everyone is on the same page. Participants will not enter each design session blindly; instead, they will have a clear idea and expectations for each design session. This strategy was informed by the CAMH Lived Experience Advisory Group meeting in February 2023. Email progress updates will be used to communicate with participants (ie, members of the co-design team) between sessions. We will also inform participants about how we have incorporated their feedback into the implementation strategies. All participants will have access to web-based or telephone support between meetings to address any questions or concerns. The following sections detail the goals of each co-design session.

##### Predesign Plan

When designing an implementation strategy, it is important to consider the full range of available intervention options [77]. The BCW links the COM-B to the full range of 9 “intervention functions” (ie, strategy types) that can change behavior [77]. Using the BCW, we will identify a full range of strategies that match to the determinants of behavior change based on phase 1 outputs. In addition, we will prepare a list of BCTs that match to the selected strategies.

##### Co-Design Session 1

There will be an opportunity to get to know the team members. Then, the findings from phase 1 (ie, the list of barriers and facilitators mapped to the COM-B) will be presented with visual aids. As a team, we will confirm the target behavior and then identify which barriers are modifiable and which facilitators can be leveraged. Activities will be used to generate insights into what change is needed to integrate the Hope app into the normal workflow. Ideas will be sought regarding *when* (ie, timing within discharge), *who* (eg, psychiatrists, nurses, or both), and *how* clinicians should deliver the Hope app.

##### Co-Design Session 2

A summary of the findings from session 1 will be shared with the design team before the meeting. During the design session, prematched strategies and possible content or contents of the implementation strategies (eg, BCTs) will be presented. We will come up with lay language alternatives to BCTs to enable better understanding. The design team’s views on the relevance and importance of BCTs will then be asked. Activities using both voting and discussion will focus on prioritizing the content and modes of delivery. The design team will then be asked to identify and discuss potential challenges regarding feasibility associated with the selected content or contents and mode or modes. The discussion will be guided by the BCW APEASE criteria, with a strong emphasis on the *equity* domain of APEASE.

##### Co-Design Session 3

The prototype of the implementation strategies will be developed based on the findings from session 2. This will be presented before the meeting and during the co-design session for review following the reporting criteria by Proctor et al [110] for describing implementation strategies. We will also provide a visual representation of how strategies intend to work, linking anticipated behavioral outcomes with mechanisms of action (ie, underlying theoretical assumptions and BCTs that will target the barriers). We will then discuss the operationalization of the strategies and their usability.

##### Co-Design Session 4

During the last session, we will present the revised implementation strategies along with the operation plan. We will present a visual storyboard, from triage to discharge, and illustrate how a patient is expected to receive the SPI via the Hope app. Specifically, we will highlight how, when, and by whom the Hope app will be delivered. The design team will have the opportunity to provide additional feedback.

#### Data Collection

All design sessions will be audio recorded, transcribed verbatim, and deidentified. There is great flexibility in the ways in which people approach co-design [111]. We will use a range of activities (eg, think-aloud and voting) to engage participants and provide them with interactive ways to develop and share ideas. We will have a mock-up trial with the internal research team and receive feedback from the ED medical head before finalizing the activities. Other data may include participants’ sticky notes of ideas or web-based comments. A cofacilitator will take notes and capture details of the design process (eg, decisional conflicts and group dynamics). In addition, at the end of each design session, we will collect data to evaluate and monitor the experience and engagement of participants. The evaluation questions will consist of Likert scales adapted from the Public and Patient Engagement Evaluation Tool [112]. The questions will ask participants to rate their experience and self-perceived engagement.

#### Data Analysis

The analysis of transcripts and notes will be pragmatic, focusing on the specific design goals outlined in Figure 1. At least 2 researchers will use NVivo [104] to independently analyze transcripts using directed content analysis [103] to categorize design considerations using the BCW as a codebook. Design session notes made by a cofacilitator will supplement data analysis to provide contextual information. We will meet regularly to resolve any coding discrepancies through discussion. In addition, we plan to do a recap of the previous design session at the start of each design session for member-checking purposes [105]. We will review the evaluation data between each design session to monitor and assess whether any changes are required to improve engagement. We will report engagement evaluation data using descriptive statistics. Outputs from phase 2 include discrete implementation strategies that not only match the determinants of implementation from phase 1 but are also reflective of inputs from several knowledge user groups.

#### Anticipated Challenges and Mitigation Strategies

There may be concerns about placing service users, family members, and clinicians in the same group as service users may feel uncomfortable speaking up. However, previous research has shown that forming a heterogeneous co-design team with patients and clinicians can be done well [113-116]. Furthermore, the CAMH Lived Experience Advisory Group supported this as an effective approach for reaching a consensus. The principal investigator of this work (HDS) also has previous co-design experience as a clinician participant in a mixed group with youth and parents [113]. To provide a trusting and safe space for all participants to share their ideas and speak respectfully to each other, the project lead (HDS) and cofacilitator will put great effort into practicing transparent dialogue and power sharing, developing a common language and avoiding medical jargon, and fostering mutual respect and making it clear to knowledge user groups that their different expertise is equally valued [117]. The Lived Experience Advisory Group also highlighted the importance of providing orientation to the co-design team members, ensuring that everyone is on the same page and has a clear understanding of the research agenda. Research team members of phase 2 will also practice humility and self-reflexivity, which are facilitators for conducting collaborative research [118]. Finally, we will be collecting progress outcomes on engagement to identify whether modifications are needed for subsequent co-design sessions.

#### Trustworthiness

There are four criteria for ensuring the trustworthiness of qualitative studies (phase 1-2): (1) credibility, (2) confirmability, (3) dependability, and (4) transferability. Credibility will be achieved by representing research findings drawn from the participants’ original data and accurate interpretations of the participants’ original views [119]. We will select and show representative quotations from the interviews when reporting the findings. Member checking will further ensure credibility. Confirmability is about ensuring that findings are the result of the experiences and ideas of the participants rather than the characteristics and preferences of the investigator [119]. Therefore, data analysis will involve ≥2 researchers. To demonstrate dependability, all processes and rationales within the study will be recorded and reported in detail [120]. Such documentation will include operational detail regarding sampling, data collection, process logs, memos, and reflexive notes. We will strive for detailed reporting using the COREQ (Consolidated Criteria for Reporting Qualitative Research) checklist [121] for phase 1 and the GRIPP (Guidance for Reporting Involvement of Patients and the Public) reporting checklist [122] for the co-design phase 2. Any deviations from the protocol because of the iterative nature of qualitative studies will be transparently described in the full report. Transferability may be limited as this study takes place in 1 clinical setting (ie, the CAMH ED) given its unique characteristics as a stand-alone psychiatric ED. However, one approach to enhance transferability is to provide detailed contextual information about the study and the setting, so called “sending context” to readers [120]. It is suggested to provide a clear description of culture and context, selection and characteristics of participants, data collection, and process of analysis [119]. Therefore, readers of the study report can make judicious transfers and inferences and apply the study findings to other settings.

### Ethical Considerations

Ethics approval for phase 1 was obtained from the Research Ethics Board of the CAMH (REB 2023/078). Recruitment and data collection in phase 1 have started. All participants are provided with both written and oral information about the study, which includes details on the voluntary nature of participation and their ability to ask questions or withdraw from the study at any time without having to provide reasons. After reviewing the study procedures, all participants provide informed consent through an electronic platform (ie, REDCap) where they provide their signature before participating in audio recording. The study data will be deidentified before publication. Clinicians participating in phase 1 will receive a CAD $50 (US $36.44) e–gift card of their choice as compensation for their time and knowledge. At the end of the phase 1 interviews, clinicians are invited to express their interest in participating in phase 2. Ethics approval for phase 2 is underway at the time of writing and will be obtained from the Research Ethics Board of the CAMH.

## Results

The protocol presents the entire structure of the research project involving 2 sequential studies, with the first phase feeding into the next. Ethics approval from the CAMH for phase 1 has been obtained, and we expect to complete recruitment and data collection for phase 1 no later than December 2023. We anticipate that each phase will last a maximum of 1 year.

## Discussion

### Expected Findings

In the end, we will have developed theory-informed implementation strategies to support CAMH ED clinicians’ uptake and subsequent delivery of the Hope app to patients. These developed strategies will facilitate the implementation of the Hope app by addressing barriers and leveraging facilitators in the local context, thereby maximizing SPI delivery. The co-design approach is expected to ensure that strategies are targeted to meet the needs of knowledge users (ie, both patients and clinicians) and are feasible, acceptable, and appropriate for the CAMH ED. In addition, the strategies driven by the BCW will change clinicians’ behavior and be compatible with their routine clinical workflow.

The patients’ behavior after ED discharge is beyond the scope of our study and also beyond the control of the ED clinical team. However, we recognize that it is another significant contributor to the effective use of the SPI. Changes in clinicians’ behavior in the ED can trigger a chain of behavior changes among patients and families. If patients and their families receive the safety plan through the Hope app in the ED, along with instructions on how to use it, as a result of successful behavior change within the ED team, this could hypothetically lead to changes in the behavior of patients and their families. After being discharged from the ED, patients may incorporate the app into their self-care routine, engage with its features, and review their safety plan and participate in self-reflection. However, if patients do not use their safety plan on their phones after ED discharge, it will not generate its intended benefits, and they may return to the ED in the event of another crisis (eg, a suicide attempt). Nevertheless, changes in patients’ and families’ behavior are beyond the scope of our study and also outside the control of the ED team. This falls into another important area for future exploration related to user engagement in health informatics.

### Strengths and Limitations

The systematic and replicable reporting of the behavioral mechanisms of action behind the implementation strategies is a strength of this study. Furthermore, the proposed co-design process is highly collaborative. The design team members will work together in equal and active partnership. In addition, this research project can provide recommendations on how to involve multiple knowledge user groups and offer insights into how the collaborative approaches can be used and adapted to other areas within the health informatics literature. However, this study does not intend to generate generalizable implementation strategies for an app. Instead, the findings can be adapted to different contexts, and we will take the necessary steps to ensure the transferability of this work outlined in the Trustworthiness section. We would also like to acknowledge the limitations of this study concerning digital equity. Digital equity encompasses more than the acquisition of digital literacy skills and ensuring affordability. This study does not address structural factors contributing to the digital divide, such as socioeconomic status and place of residence. Moreover, it cannot resolve the access divide, which reinforces our decision to keep the paper-based SPI in the ED as an option. Although we recognize that digital equity surpasses digital literacy, it is also important to acknowledge that equity must extend beyond ensuring affordability; even with access to technologies, the reported outcomes of using health technologies have been inconsistent [64]. In our study, we prioritize digital literacy and the engagement of various knowledge user groups in the design process, aiming to alleviate potential challenges associated with the app’s use among individuals with limited digital skills. Finally, co-design team members will be self-selected, often motivated individuals, and their views may not be representative of diverse ED service users and clinicians at the CAMH.

### Conclusions

This proposed work will contribute to the current literature by addressing several identified gaps. First, this work will contribute to the identified knowledge gap regarding the contextual implementation characteristics of ICTs for suicide prevention in clinical settings. Using the BCW, this work will also tease apart the nuanced influences of behavior change leading to ICT implementation. Second, this theory-informed and collaborative work will fill the methodological gap regarding nonrigorous implementation research for mental health apps.

## Appendix

Multimedia Appendix 1 Hope app content.

Multimedia Appendix 2 Semistructured interview guide for phase 1.

## Abbreviations

APEASE: Affordability, Practicability, Effectiveness, Acceptability, Safety, and Equity
BCT: behavior change technique
BCW: Behavior Change Wheel
CAMH: Centre for Addiction and Mental Health
COM-B: Capability, Opportunity, and Motivation–Behavior
COREQ: Consolidated Criteria for Reporting Qualitative Research
ED: emergency department
GRIPP: Guidance for Reporting Involvement of Patients and the Public
ICT: information and communications technology
IKT: integrated knowledge translation
KTA: Knowledge-to-Action
REDCap: Research Electronic Data Capture
SPI: safety planning intervention
TDF: Theoretical Domains Framework

## Data Availability

All data generated and analyzed in this study will not be publicly available in accordance with the Centre for Addiction and Mental Health ethical review guidelines as all data are securely stored on an internal server. However, they can be obtained from the corresponding author upon reasonable request.
