# Supplementary material for: Designing Implementation Strategies for a Digital Suicide Safety Planning Intervention in a Psychiatric Emergency Department: Protocol for a Multimethod Research Project
Source: JMIR Res Protoc. 2023 Nov 9;12:e50643. doi: 10.2196/50643 (PMC10667981; doi:10.2196/50643)
Supplement: Multimedia Appendix 1 [file resprot_v12i1e50643_app1.docx]

Multimedia Appendix 1. Hope app content

| **Home page**  **Contents:**   - Safety Plan - Community Resources (curated) - Wellness Activities (e.g., Journaling, Grounding technique) - General Information (e.g., Causes and risk factors for suicide) - Crisis Line | **Core Component**: Safety Plan  Note: Safety plan includes 5 items | Example View 1  Item 1: My Warning Signs | Example View 2  Item 2: My Reasons for Living |
| --- | --- | --- | --- |
| 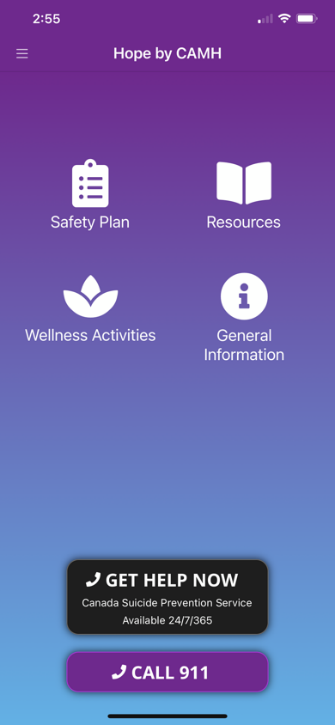 | 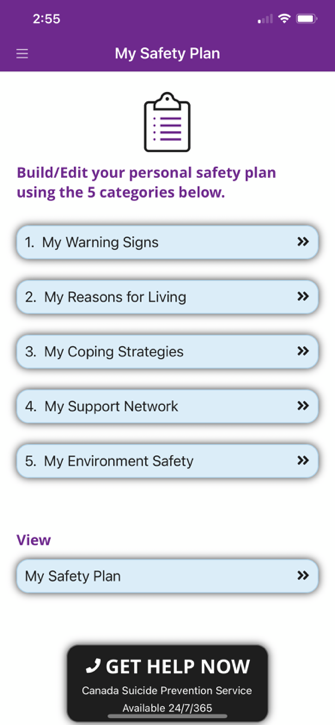 | 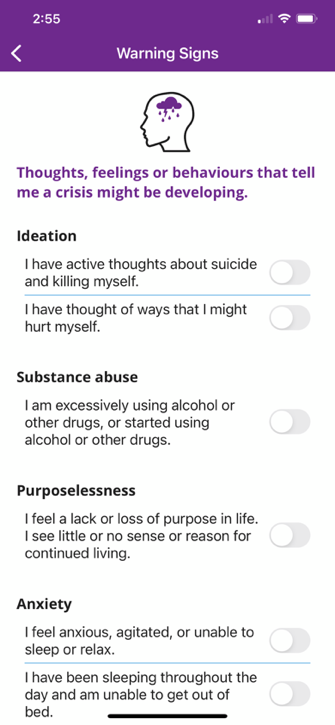 | 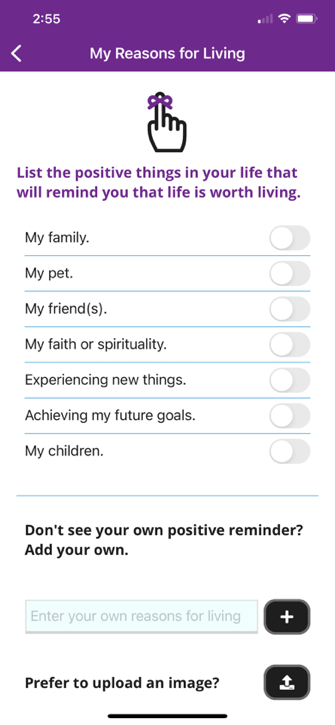 |
